# Using a Community-Based Participatory Research Approach to Study the Mental Health of Older Adults with a Refugee Life Experience

**DOI:** 10.3390/ijerph22081303

**Published:** 2025-08-20

**Authors:** Rochelle L. Frounfelker, Puja Thapa, Tej Mishra

**Affiliations:** 1 Department of Population Health, College of Health, Lehigh University, Bethlehem, PA 18015, USA; put224@lehigh.edu; 2 Bhutanese Community in Harrisburg, Harrisburg, PA 17112, USA; tmishra@bcharrisburgpa.org

**Keywords:** CBPR, older adults, mental health, Bhutanese, refugees, migrants

## Abstract

Older adults with a refugee life experience have a disproportionate burden of mental health problems compared to non-refugee aging populations. Community-based participatory research (CBPR) is a promising approach to identify the challenges and strengths of older refugee adults and identify solutions to mental health disparities. We present a case study of one such CBPR program of research with older ethnic Nepali Bhutanese adults resettled in North America, Project Bhalakushari. We highlight the many opportunities to integrate older adults and members of the broader Bhutanese community in activities throughout the research process, starting with forming academic–community partnerships, identifying the needs and strengths of the community, conducting research, and sharing study findings with a broad audience of community members, health practitioners, policy-makers, and academics. Our case study emphasizes that a successful, culturally informed partnership should consider the involvement of the entire community, regardless of age, in the initiative to secure buy-in and support and maximize the positive impact of the work. We identify concrete strategies to overcome challenges specific to conducting research, with a focus on recruitment, outreach, and data collection.

## 1. Introduction

Migration is a social determinant of health, with migrants frequently having limited social and economic opportunities and lack of access to appropriate health care [1,2]. Within the larger migrant population, forced displacement due to conflict and persecution has particularly negative effects on mental health [3,4,5]. In the past decade, the forcibly displaced population increased at an alarming rate worldwide, with the most recent data from the UN High Commissioner for Refugees (UNHCR) indicating that the number (predominantly from the Global South) of civilians displaced from their homes reached over 123 million in 2024, with refugees comprising about 43 million of that total [6].

UNHCR estimates that older persons make up about 8.5% of the total population of those who are forcibly displaced [7]. Unfortunately, this subgroup oftentimes receives limited attention from organizations and governments [8] despite facing unique challenges in the processes of both aging and integration [9]. Compared to other older adults, older refugees have a disproportionate burden of challenges related to past traumas, language barriers, family stress, social support, employment, and accessing health care [10,11,12]. These challenges and stressors, in turn, are associated with poor mental health. There are mental health disparities between older refugees compared to others in the same age group in host countries of resettlement [13,14,15,16,17].

### 1.1. Older Adults and Mental Health Research

There is growing interest in investigating the mental health of older adults with refugee life experiences, with the goal of developing and/or adapting evidence-based mental health interventions that can address the unique needs of this group. A significant challenge to this endeavor is engaging older adults in research to build an evidence base for addressing these needs. Specific to the US, researchers focused on barriers in reaching and engaging older adults from minoritized, vulnerable populations. This is particularly problematic, as subgroups based on race, ethnicity, and immigration status are both disproportionately underrepresented in research while also being disproportionately burdened by negative health outcomes [18,19,20]. Researchers identified a range of micro- to macro-level barriers to participation, including a lack of trust, family issues, communication barriers, knowledge and understanding of research goals, views of health and illness, and socioeconomic status [20,21,22].

To date, a variety of models and strategies have been proposed to promote participation among aging populations, with approaches situated along the continuum of community engagement in research [23,24]. The MyAlliance for Brain Health developed a three-arm recruitment plan, engaging primary care providers, patient and family networks, and community organizations in their protocol [25]. The culturally informed community engagement research (CI-CER) model emphasizes using culturally informed research methods and more inclusive engagement strategies, such as community–science partnership boards, to promote participation [26]. Walter et al. [27] articulate that aging research conducted in partnership with the public is critical, with opportunities for engagement taking place throughout the research cycle.

### 1.2. Community-Based Participatory Research

Some researchers explicitly identified the need for more community-based participatory research (CBPR) or participatory action research (PAR) initiatives with older adults [28,29]. CBPR developed as a research approach over the past 25–30 years, which is distinct in its focus on including community members throughout the research process. Wallerstein et al. [30] (p.3) provide this definition: “CBPR embraces collaborative efforts among community, academic, and other stakeholders who gather and use research and data to build on the strengths and priorities of the community for multilevel strategies to improve health and social equity.” There are 10 key principles to CBPR, including an emphasis on local community capacity building, being a co-learning process between all stakeholders, achieving balance between research and action, and an empowering process for participants and community members [30]. CBPR is not a method; it is rather an orientation and approach to the research process that focuses on equitable research partnerships and social transformation [31].

A CBPR approach has been used with vulnerable populations including refugees and older adults, and for research focused on the topic of mental health. A review of CBPR with refugees identified 14 studies, ranging from topics as diverse as mental health, sexual and reproductive health, to cardiovascular disease [32]. Findings indicate a lack of involvement of refugees in some stages of the research process, such as obtaining funding and participating in data analysis [32]. In terms of older adults, CBPR is overall underdeveloped, with a focus needed on building trust, training older adults for their roles in research, and addressing power differentials between researchers and older adults [28,33]. Specific to mental health, a systematic review of CBPR applied to mental health intervention research found that, out of 14 studies, interventions had a positive impact on mental well-being outcomes, but noted that adherence to CBPR principles in the research process was overall low [34].

With its focus on equitable partnerships and building trust between stakeholders, CBPR holds great promise for mental health research with older migrant adults. To date, there has been limited exploration on the application of CBPR with this population. We address this gap and present a case study of an ongoing CBPR program of research on mental health of older adults with a refugee life experience in North America, Project Bhalakushari.

## 2. Materials and Methods

### 2.1. Bhutanese with a Refugee Life Experience

Project Bhalakushari centers on the refugee life experience of ethnic Nepalis forced to leave their home country of Bhutan in the late 1980s and early 90s due to government persecution [35]. Over 100,000 refugees fled from Bhutan to Nepal, living in refugee camps for 15–20 years before third country resettlement began in 2008 [36,37]. Since that time, over 90,000 ethnic Nepali Bhutanese relocated to the United States (US) [38]. In the early 2010s, the Centers for Disease Control conducted a study on Bhutanese refugees, given their disproportionately high rate of suicide compared to the general US population [39]. There has been thoughtful work on the mental health of resettled Bhutanese in the US, ranging from the topic of suicide [40,41,42] and attention to the needs of the population throughout the course of life, from youth to older adults [43,44]. In our work, we intentionally use the term “Bhutanese with a refugee life experience,” as opposed to “Bhutanese refugees” given that the vast majority of the population in the US are now citizens, and the label of “refugee” can be stigmatizing.

### 2.2. Approach

Project Bhalakushari began in 2017 with a pilot project that included 190 older Bhutanese with a refugee life experience and 10 health care providers. This convergent mixed methods study included a quantitative cross-sectional survey of all 190 older adults and in-depth one-on-one qualitative interviews with 41 older adults and 10 formal and informal health care providers who work with older adults in the community. Our current phase of Project Bhalakushari began in 2024 and will continue until 2029. This sequential mixed method study includes first exploratory qualitative one-on-one interviews with 50 individuals (20 older adults, 20 caregivers of older adults, and 10 health care providers) to examine the construct of social support within the context of Bhutanese culture. This qualitative research will inform the second, longitudinal part of the study, in which we will enroll dyads of 200 older Bhutanese and 200 people they identify as caregivers. These 400 participants will complete a quantitative survey at three time points, each one year apart. Additional qualitative, one-on-one interviews will be conducted with a subset of 40 caregivers in this sample to explore the caregiving experience among Bhutanese.

We map out the history of the Project Bhalakushari research program, ways in which the community has been involved in the research, and identify challenges, opportunities, and lessons learned. We walk through all phases of the research process, from formation of the partnership, carrying out research, to maintaining and sustaining the CBPR partnership. The authors write from their perspectives as an outside academic co-leading the research (first author), a Nepali research scientist currently conducting outreach and data collection in the field (second author), and an ethnic Nepali Bhutanese community leader, research co-leader, and public health practitioner (last author). See Table 1 for a review of challenges and recommendations.

## 3. Results

### 3.1. CBPR Partnership Formation and Identification of the Problem

The CBPR partnership for Project Bhalakushari came out of an existing CBPR program of research initiated by the Bhutanese community in Massachusetts and researchers at Harvard Chan School of Public Health in the early 2010s. This research focused on adapting and piloting an existing evidence-based mental health intervention designed to promote the mental health of youth and overall family functioning of Bhutanese families in the greater Boston area and Western Massachusetts [45,46,47]. The first and last authors were members of this research team starting in 2013, and they helped carry out formative work, including conducting a community needs assessment, identifying relevant ethnic Nepali mental health syndromes and terms, and adapting the Family Strengthening Intervention to meet the needs of refugee families [45,47,48].

During Community Advisory Board meetings and other more informal community conversations and gatherings, the Bhutanese articulated a need for and interest in understanding the issues impacting older members of their community. Appreciative of the important work being carried out with youth and families, they asserted that older generations were also struggling to adapt to life in the US and experiencing mental health challenges. In 2017, members of the research–community partnership team successfully applied for pilot funding to carry out formative research on the topic. The aims of this mixed methods research were threefold: (1) qualitatively explore mental health care needs, views on mental health and well-being, and risk and protective factors for the mental health of older Bhutanese adults; (2) quantitatively examine the relationship between exposure to pre-resettlement stressors, post-resettlement stressors, and mental health among aging Bhutanese; and (3) integrate qualitative and quantitative findings to identify psychosocial intervention activities for older adults that can inform future research. The core research team, composed of academic and community members, decided on the name Project Bhalakushari for community-facing work, as bhalakushari is a Nepali word meaning a casual conversation, something familiar and accessible to older adults. At the time of its formation, Project Bhalakushari was a partnership between researchers at Harvard Chan School of Public Health and the Bhutanese Society of Western Massachusetts, a self-help community organization.

Since 2017, the CBPR partnership for Project Bhalakushari traveled as the core research team members left Massachusetts and reunited in Pennsylvania in 2022. This is in part due to patterns of secondary migration within the US Bhutanese community, with significant growth in the greater Harrisburg region in central PA. There is currently a formal partnership between researchers at Lehigh University and the self-help Bhutanese Community in Harrisburg (BCH) organization, with project leadership composed of people at both institutions, including the Community Advisory Board (CAB). BCH advocates and for and represents the interests of an estimated 40,000 ethnic Nepali Bhutanese with a refugee life experience living in the region.

The CAB is an inseparable part of our CBPR approach in Project Bhalakushari. To date, we engaged around 10 community leaders. The CAB represents notable and experienced community leaders from various backgrounds, including education, literature, health, homecare, nonprofit, and more. These diverse insights contributed to refining Project Bhalakushari’s study design, tools and instruments, and outreach strategies.

Bringing together a diverse group of leaders from different backgrounds provided varied perspectives while designing and conducting research; however, it may also present challenges, as each member may hold established, preconceived notions about what is important to the community and how the research should be conducted. When faced with such challenges, the research team reassures CAB members that the research priorities are evidence-based and originated within the community by emphasizing the CBPR approach and the project’s history. Furthermore, we focus on creating a collaborative environment by encouraging academic researchers, community leaders, and community RAs from diverse ethnic, educational, and social backgrounds to engage in mutual respect, shared responsibility, and collective efforts toward a common goal throughout the process. We clearly explain expectations from the beginning and consistently remind everyone of the collaborative norms.

Another challenge that may arise is consistently engaging CAB leaders due to their demanding personal and professional schedules, a challenge observed more frequently among women leaders. Gender norms may manifest as imposter syndrome among women community leaders involved in the CAB, leading them to doubt their abilities and downplay their accomplishments. This can negatively impact women’s representation in the CAB. To ensure consistent and meaningful participation, we support engagement by adapting to participants’ availability and responsibilities and by scheduling follow-up calls with leaders who are unable to attend meetings. This approach helps keep them updated and allows for their feedback on study tools and design. Additionally, to encourage women’s participation, we emphasize that their meaningful representation is essential for a holistic approach and to ensure that women’s perspectives are reflected in the advisory process.

### 3.2. Identifying Community Strengths, Dynamics, and Priority Issues

When we speak of engaging in CBPR with older Bhutanese adults, our definition of community is, importantly, inclusive of people who identify as ethnic Nepali Bhutanese of all ages. Other CBPR aging research focused primarily on the unit of ‘the community’ being composed of older adults [28,33], and emphasized the engagement of people in this age group throughout the research process. This is certainly important, and something we consider in our work. However, given the cultural context and dynamics of the ethnic Nepali Bhutanese community, our partnership emphasized the importance of building an equitable partnership with the larger sociocultural group. In fact, we believe that focusing exclusively on older adults would not be culturally appropriate and would be off-putting for many people in this very collectivist community.

The strengths and dynamics of any community change over time and may vary by geographic location, and this is a particularly salient point when working with refugee populations that undergo rapid changes during the refugee life trajectory. Although some of the mental health concerns of the recently resettled Bhutanese community in Massachusetts in 2013 persisted, such as the topic of suicide and suicide prevention, others changed. For instance, there is an urgent need to investigate the intersection of mental health and substance misuse, as well as other behavioral problems, such as gambling addiction, in central PA. The very process of understanding these needs also changes over time, as conducting a needs assessment with a community of several hundred Bhutanese families in New England looks very different from understanding the needs of a more geographically diverse population in central PA with 40,000 members.

As mentioned above, Project Bhalakushari came out of existing CBPR work with the Bhutanese community in Massachusetts in which an in-depth needs assessment was conducted to understand the strengths, challenges, and needs of the community in relationship to children and families [45]. Our pilot work provided more detailed information on the needs and strengths of older adults and current mental health issues, including PTSD, depression, and anxiety [49,50,51]. We find that the health of older adults is still an important issue today, but must be situated within the context of new challenges and priorities. For instance, cultural preservation and documenting the history of ethnic Nepali persecution in Bhutan and the migration journey is a much more urgent concern in the community now than it was ten years ago given the aging of adults with lived experience of persecution; as such, we see Project Bhalakushari as a mechanism to achieve these goals as we conduct qualitative interviews and gather the life narratives of older adults that can become part of a larger archive of oral history. Including younger community members as part of our data collection and analysis team also serves this purpose, as younger generations are exposed to and learn more about the experiences of their elders, which is infrequently talked about in more informal settings.

### 3.3. Designing and Conducting Research

#### 3.3.1. Grant Proposal Writing and Study Design

Our grant proposal writing process is led by academic researchers, with the study principal investigator drafting material that is then reviewed by community partners for input and feedback on feasibility and cultural appropriateness. We believe in complete transparency when developing budgets and allocating resources between academic and community stakeholder groups to minimize frustration and resentment over how money is being spent on either side. Study design issues, such as research methods, are discussed and decided collectively. For example, community members involved in the decision-making process for our pilot grant application in 2017 included those with previous CBPR experience in mixed methods research. As such, there was interest in using a mixed methods study design and collecting both qualitative and quantitative data with older adults. Ultimately, the study used a convergent mixed methods study design to achieve the overarching goals of the work. In our current study, we are once again using a mixed methods study design, with longitudinal quantitative data being collected over multiple time points to detect changes in mental health over time.

The team makes group decisions about sampling schemes for our work, such as stratifying participants by age and gender to ensure representation from people with a wide range of experiences. The minimum age for inclusion in our studies is 50, based on the age agreed upon by community leaders as indicating older adult status. There are ongoing discussions on this subject, as perceptions of old age are rapidly changing in the community, with the number of adults living over the age of 75 on the rise. For instance, our most recent CAB meeting included brainstorming strategies to promote study enrollment among adults under age 65. For our pilot work, researchers and community members also decided to enroll health care providers for qualitative interviews, using a broad definition of “provider” that included informal health care practitioners, such as religious leaders and traditional healers, given their importance among older adults in the community.

#### 3.3.2. Study Tools and Instruments

Bhutanese community members are critical in selecting and developing study tools and instruments. Specific to quantitative assessment batteries, the study PI uses scientific expertise to identify assessment instruments that are appropriate, reliable, and valid for use with older adults. However, there is much discussion within the research team and CAB members on important constructs that should be assessed in order to more fully understand the mental health of aging community members. For instance, Bhutanese team members, including those aged 50 and over, suggested including a measure related to religiosity in our assessment battery. The rationale, they argued, was that trying to understand the psychosocial well-being of older adults without measuring religiosity was comparable to “trying to make curry without salt”.

A key aim of our work is to understand the pathways between exposure to traumas in Bhutan prior to fleeing the country, life in refugee camps in Nepal, current stressors in resettlement, and mental health. As such, our assessment battery includes a detailed index of traumatic events participants may have experienced prior to arrival in the US. The research team reviewed existing trauma inventories frequently used with refugees, and Bhutanese members were asked to generate a list of additional events that are historically and culturally relevant for their refugee life experience. Examples of new items generated for experiences in Bhutan included “Were you ever forced to stop speaking Nepali?” and “Were you ever forced to sign documents against your will?” For life in Nepal, additional questions included “Did you experience a widespread fire that destroyed homes?” and “Did you ever experience discrimination by local communities because of your refugee identity?” Together, those with a lived experience reviewed these items and selected those deemed most important for inclusion in our final assessment battery.

Community members also contribute to the development and refinement of qualitative interview guides. In our pilot study, social support was an important moderator of the relationship between past traumas and stressors and current mental health. Our current work is using qualitative methods to investigate social support more in depth, with interviews on the topic being conducted with older adults, their loved ones, and health care providers. Bhutanese team members generated interview guide questions that they felt were relevant for understanding the experience of older adults. For example, Bhutanese members generated the questions “What are the responsibilities of others to take care of you?” and “Are these responsibilities being met?” for older adults because of cultural expectations around caregiving in the community.

#### 3.3.3. Data Collection

Our data collection process is conducted by community research assistants (RAs), under the leadership of the second author. Below, we highlight issues including RA capacity building, community outreach, and participant recruitment. In adopting the CBPR approach, the team encounters various challenges stemming from socio-political, cultural, geographical, and demographic factors. However, each challenge is addressed through concrete strategies developed by our interdisciplinary research team, which brings together expertise in mental health and community-based research, as well as lived experience and cultural knowledge.

In alignment with the CBPR approach, bilingual English–Nepali speaking community members are employed as RAs and conduct participant recruitment, field data collection (conducting qualitative interviews and quantitative surveys), and other logistic activities. Qualifications for being an RA include fluency in reading and speaking both English and Nepali; there are no educational requirements for the position. One challenge is identifying qualified RAs from the younger generation in the community. While many younger members speak Nepali and are eager to be involved in this work, they often lack the communication skills needed to effectively engage with older community members. To address this, we train local individuals who already have strong connections within the community, fluency in the language, and experience interacting with older adults. Our team is composed of community members from different generations. By equipping them with foundational research skills, we ensure a community-centered process and strengthen local voices in the process. All RAs complete research ethics training and learn about research and the research process through didactic and experiential learning. There is intensive research skill building prior to starting outreach and recruitment work.

Community outreach is conducted by the team using multiple strategies, including door-to-door outreach, phone recruitment, word-of-mouth referrals, and social media recruitment. Community leaders from the CAB, and other community partners, play a central role in identifying potential participants and developing outreach strategies. RAs also engage in community gatherings, including religious services and social meetings at local churches and temples, to introduce the study, recruit participants, and provide insights into the research process.

Academic research is a fairly new concept for the community, especially among older adults. One of the challenges we face is conveying the purpose and structure of research in an accessible and relatable way. In response, the team takes time to explain how research interviews differ from traditional journalistic audio/video content that participants may have previously been exposed to. We highlight the confidentiality of the interviews, the community-driven nature of the project, the voluntary aspect of participation, and the long-term value—such as building a historical archive for future generations. Once participants understand these aspects and the uniqueness of academic research, they are frequently, but not always, more interested in participating.

Bhutanese community members, especially those aged 50 and above, experienced trauma—having fled their homeland due to ethnic cleansing, lived in refugee camps in Nepal for decades, and then experiencing third country resettlement. This history often left them with lingering fears of being surveilled or deported. Considering this existing fear, we approach the research with deep sensitivity to intergenerational trauma. We are transparent, build trust over time, and reinforce the confidentiality of information. Most importantly, we express our shared concern for community safety, validating their fears while offering reassurance through clear, honest communication.

Another challenge we face is that older adults often doubt their ability or “expertise” to share personal stories due to a lack of formal education, often leading to hesitation or underestimation of the value and importance of their lived experiences. We are often met with initial responses such as, “but I wouldn’t know what to say,” “I am not educated,” or “I won’t be able to answer the way you would want me to.” To address this, we emphasize that the interview is centered on their experiences, with no right or wrong answers. We frame it as bhalakushari to ease pressure and formality.

Once initial interest is established through various forms of outreach, the research team follows up with eligible individuals via phone or in-person visits to schedule interviews. Interviews are conducted in person at private, participant-preferred locations—most often in participants’ homes or at a community organization office space—to ensure confidentiality.

In communities such as the Bhutanese, gaining participation often requires first speaking with family members, as older adults may have limited autonomy in decision-making. The limited autonomy also posed initial challenges in maintaining privacy during the interview, as sometimes younger family members want to hear what their relatives are saying. To address this, we first reach out to the head of the family to address any doubts or concerns. We explain the importance of privacy to ensure confidentiality. Following this, we speak directly with older adults to explain the research purpose and process. In many families, informed consent is obtained in the presence of family, clarifying that participants are free to share or withhold interview details with others after it is completed.

Once participants are recruited and interviews scheduled, we encounter some rescheduling challenges as a result of older adults’ health conditions. To address this, we adopt a patient and persistent approach. RAs check-in compassionately and offer rescheduling options, reinforcing our long-term goal of building sustainable, trust-based relationships aligned with CBPR principles. Adopting a CBPR framework during the data collection process allows us to collect data of the community, by the community, and for the community. The challenges we encounter further help us refine our approach and tailor it to meet the specific needs of the community.

#### 3.3.4. Data Analysis and Interpretation of Study Findings

The lead author (and study PI) conceptualizes and leads qualitative, quantitative, and mixed methods data analysis plans. However, the process of data analysis and interpretation of study findings is carried out using a team approach, with Bhutanese members of the research team playing a critical role. Qualitative interviews are translated and transcribed by community research staff; community researchers have been trained in thematic content analysis [52] and jointly analyze data using MAXQDA 2022 mixed methods software [53]. As a community outsider, the lead author defers to Bhutanese to explain and contextualize historical events study participants speak about in interviews in order to accurately analyze and interpret data. Specific to quantitative data analysis, in addition to being a study co-investigator, the third author has advanced training and expertise in biostatistics, and led some data analysis under the guidance of the study PI. Recently, ethnic Nepali doctoral students affiliated with the team led the analysis, writing, and preparation of a manuscript for publication [54].

Academic and community partners jointly discuss and interpret study findings, with a focus on validation of these findings based on feedback from those with a lived experience. Preliminary results from our pilot work were shared with older community members, including those who participated in the study, to obtain their thoughts. We find that it is important for all team members to recognize and acknowledge their biases when it comes to interpretation of study findings, and we engage in conversations in the spirit of being respectful but expecting to be challenged about assumptions. For instance, during one discussion about study findings, Bhutanese team members pointed out to the lead author that she was interpreting the process by which older adults made meaning of their refugee life experience from a very western, Christian, and individualistic perspective, leading to potentially inaccurate conclusions.

### 3.4. Knowledge Dissemination

Desirable dissemination of research findings can vary significantly depending upon the stakeholder group. On the academic side, it is critical to share findings in peer-reviewed academic publications, professional and academic conferences, and in applications for future funding opportunities. On the community side, priorities include sharing information with the larger Bhutanese community, local health care service providers, local government officials, and others who are in a position to fund and develop services that address the needs of older adults. All of our peer-reviewed publications are co-authored by both academic and community partners; when funding allows, academic–community teams co-present study findings at conferences and guest lectures at academic institutions. Specific to our pilot work, study findings were shared with local service providers and community members; one outcome of this was a community health center implementing weekly support groups for older Bhutanese adults, where they integrated psychoeducation on important health topics. Unfortunately, the COVID-19 pandemic prevented more widespread sharing and transformation of our results into concrete policy and service changes. It is reasonable that community partners are eager for research to be translated into action, and we are actively exploring creative and meaningful ways to disseminate knowledge in a way that will have a meaningful impact in our current work.

### 3.5. Maintaining, Sustaining, Evaluating CBPR Partnerships

Project Bhalakushari is now in its eighth year, and we are hopeful that it will continue and move forward. Our goal is to use findings from our current work to apply for funding to adapt and deliver culturally appropriate mental health services for older adults in the community. Adapting to changes, new priorities, and new challenges in the community is a work in progress. To date, we have not instituted a formal evaluation of our CBPR partnership; this will be critical as our study unfolds over the next few years.

## 4. Discussion

We highlight some of the challenges and opportunities of CBPR work centered on the mental health of older migrants, outlining how Bhutanese with a refugee life experience have been involved in research collaboration in North America for the past eight years. Our case study provides additional insight on existing literature on CBPR with older adults and the challenges of engaging older minority and immigrant populations in research initiatives. Research on CBPR and older adults highlight that there has been limited work to date on applying principles of CBPR to the health issues of aging populations; when it has been conducted, there is typically limited involvement of older adults in the research process, with exclusion in phases such as data collection and analysis in particular [28,55]. We believe that although this critique is valid, the engagement of older adults in CBPR should be understood within the context of the history and culture of the community. In our work, almost all older adults have limited education (none or at most primary school) and are illiterate in both Nepali and English. This creates real practical barriers in engagement in some aspects of the research process, without being intentionally exclusionary. This is of course not universally true, and we had CAB members and RAs who are over age 50 involved in Project Bhalakushari.

We also reflect on the idea that CBPR with older adults means focusing predominantly on the involvement of older adults in the work. In Project Bhalakushari, our definition of the “community” is not based on age, but on a shared historical and cultural background. Ironically, research on older adults in Asian immigrant communities highlights engagement challenges because older adults are typically looking to younger family members to make decisions about participating in research [56]. We find that this is true in our work, which we believe points to the necessity of framing CBPR with older adults as a larger community concern in order to obtain buy-in and support. In fact, we argue that this is one way in which our work is more broadly culturally appropriate for the age group and larger Bhutanese population [57]. Such an approach feeds into positive aspects of CBPR; researchers highlight that including older adults in CBPR has numerous benefits, such as promoting the social participation and personal growth of older adults and facilitating a deeper understanding of data and the implications of research findings [55,58]. We find that this is absolutely true, and that inclusion of people with a lived experience is imperative to draw accurate conclusions from our data and achieve research goals. Equally critical is the inclusion of younger generations from the community to promote pathways of intergenerational knowledge sharing and community building.

Finally, our case study provides insight into the challenges of trust-building and developing equitable relationships between academic and community stakeholder groups, which is core to good CBPR work. Many researchers identify lack of trust as a key barrier to engaging minority and immigrant older adults in research [20,55,57,59]. We emphasize that building and maintaining trust requires the use of a trauma-informed approach to partnerships and conducting research. Older adults with a refugee life experience, and by extension their family members and entire communities, have sometimes decades worth of experiences in which their human rights were violated. Regardless of whether or not researchers are using a CBPR approach, initiatives should be transparent, co-owned, and build on/promote the agency of those involved in all aspects of research.

## 5. Conclusions

There is a growing number of migrants globally, and there is a need to address pressing health issues that impact this population. Aging migrants, and refugees in particular, are often a forgotten subgroup and receive limited attention from researchers and policy-makers. CBPR is an approach to research that can promote the participation and engagement of older adults and the communities of which they are a part, serving to integrate the perspectives of those with a lived experience into all phases of the research process. Engaging in such work can be challenging and time-intensive, but also incredibly rewarding for everyone involved.

## Figures and Tables

**Table 1:** Challenges and recommendations for conducting CBPR with older adults with a refugee life experience.

Topic	Challenge	Recommendations and Strategies
Intergenerational challenges	Younger community members who are interested in the work may lack the linguistic skills needed to effectively engage with older adults in the community.	Identify and train local individuals who already have strong connections within the community, are fluent in the language, and have experience interacting with older adults.
Family and community dynamics	There may be restricted or limited autonomy in decision-making among older adults that determines their participation in research.	Take a family and larger community approach to engagement. Cultivate CAB membership to represent diversity in ages and important community characteristics (religion, ethnicity, etc.).
Research knowledge	Older adults may doubt their ability and knowledge to contribute meaningfully to the research due to lack of formal education. Very few individuals have prior experience participating in research and have limited understanding of what it entails.	Emphasize that the research is focused on hearing about life experiences, with no right or wrong answers. Encourage participants to share by emphasizing that their life experiences are unique, valuable, and deserve to be heard. Explain how research interviews differ from traditional journalistic audio/video content participants may have encountered in the past. Highlight key aspects of the research, including confidentiality of interviews, the community-driven nature of the project, and the voluntary nature of participation. Inform participants about the long-term value of the research—such as contributing to a historical archive that can benefit future generations.
Timelines and schedules	Older adults may have restricted movement in the community due to transportation and/or health issues. Older adults may have new and pressing health concerns that need to be addressed before participation in research.	Conduct data collection at locations convenient for participants (homes, community centers, etc.). Enlist support from family members to transport older adults to research community events and gatherings. Adopt a patient, persistent, but not pushy approach to recruitment and engagement. Offer rescheduling options and reinforce the long-term goal of building sustainable, trust-building relationships.
Gender dynamics	Gender roles and norms may make older women in particular reluctant to share information and participate in research. Gender norms may manifest as imposter syndrome among women community leaders involved in the Community Advisory Board (CAB), leading them to doubt their abilities and downplay their accomplishments. This can negatively impact women’s representation in the CAB.	Create a safe and supportive environment for interviews by pairing women research assistants with women participants. Assure participants of confidentiality to help them feel secure in sharing their experiences and perspectives. Encourage them by emphasizing that their voice as women is important and that they have full control over what they chose to share. Emphasize to the CAB women leaders that their meaningful representation is essential for a holistic approach and to ensure women’s perspectives are reflected in the advisory process. Create a supportive environment in the CAB leadership by offering flexibility, recognizing their busy professional schedules and the responsibilities they may have within their family settings.
Trauma-informed approach	Community members experienced trauma, having fled their homeland due to ethnic cleansing, living in refugee camps, and then resettlement. There are often lingering fears of being surveilled or deported. Research team members are witness to hearing about past traumatic and stressful events of older adults. Recalling traumatic life events during interviews can trigger emotional responses in both participants and research assistants; these moments can be difficult to navigate and may affect the flow and comfort of the interview process.	Practice transparency and build trust over time; reinforce confidentiality of participation. Express shared concern for community safety, validate fears, and offer reassurance through clear, honest communication. Create a supportive research team environment with opportunities to discuss and share challenging experiences. Emphasize the voluntary nature of the research, and that an individual is free to not answer questions or decide to no longer participate at any time. Incorporate training that prepares RAs for potentially emotional situations. Include role-playing and guidance on responding empathetically without overextending emotionally. Clearly inform participants that they may pause or stop the interview at any time. Have water ready before the interview begins to offer immediate comfort. Create space for RAs to debrief after interviews, allowing RAs to reflect, share experiences, and support one another, helping them process their own emotional reactions and prevent burnout.
Community Advisory Board (CAB) challenges	Engaging community leaders consistently in the Community Advisory Board (CAB) may be challenging due to their demanding personal and professional schedules. Bringing together a diverse set of leaders from different backgrounds may present challenges as each may hold established, preconceived notions of what is important to the community and how the research should be conducted.	Support engagement by adapting to CAB leaders’ availability and responsibilities and scheduling follow-up calls with leaders who are unable to attend meetings. This will help keep them updated and allow for their feedback on study tools and design. Emphasize the CBPR approach and the history of the project to reassure CAB members that the research priorities are evidence-based and originated within the community. Create a collaborative environment by encouraging academic researchers, community leaders, and community RAs from diverse ethnic, educational, and social backgrounds to engage in mutual respect, shared responsibility, and collective efforts toward a common goal throughout the process. Clearly explain expectations from the beginning and consistently remind everyone of the collaborative norms.

## Data Availability

No new data were created or analyzed in this study. Data sharing is not applicable to this article.
